# Supplementary figures and images for: Effect of genetic background on the evolution of Vancomycin-Intermediate Staphylococcus aureus (VISA)
Source: PeerJ. 2021 Jul 13;9:e11764. doi: 10.7717/peerj.11764 (PMC8284308; doi:10.7717/peerj.11764)

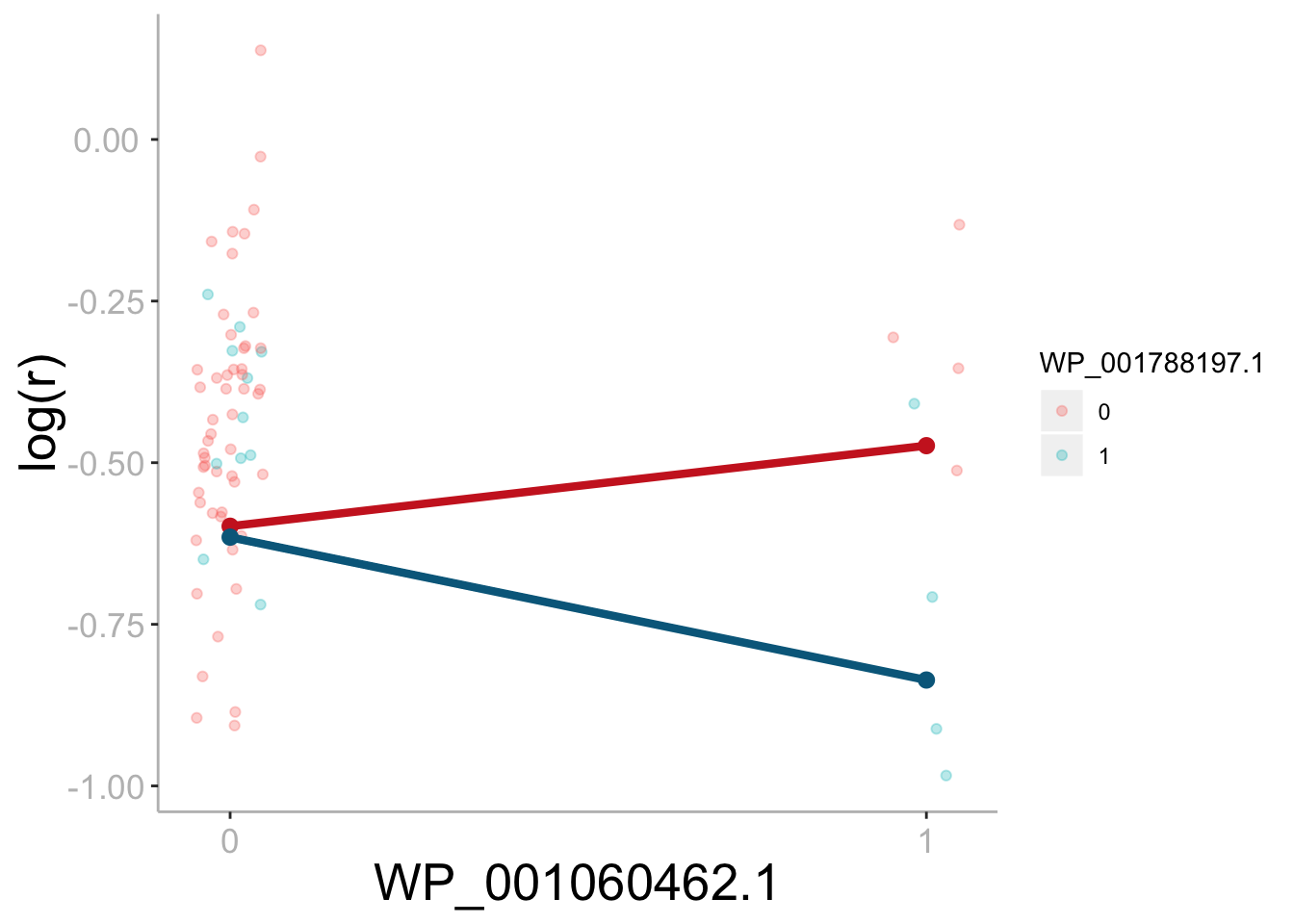

Supplement: Supplemental Information 1 — Growth rate (r) was used as a proxy for strain fitness. The difference in slope illustrates the interaction effect between sdrC and rpoC. The effect of sdrC.1 is 0.363 lower when a rpoC mutation is present (uncorrected p = 0.023). Bonferroni corrected significance threshold: ɑ = 0.00056. [file peerj-09-11764-s001.png]

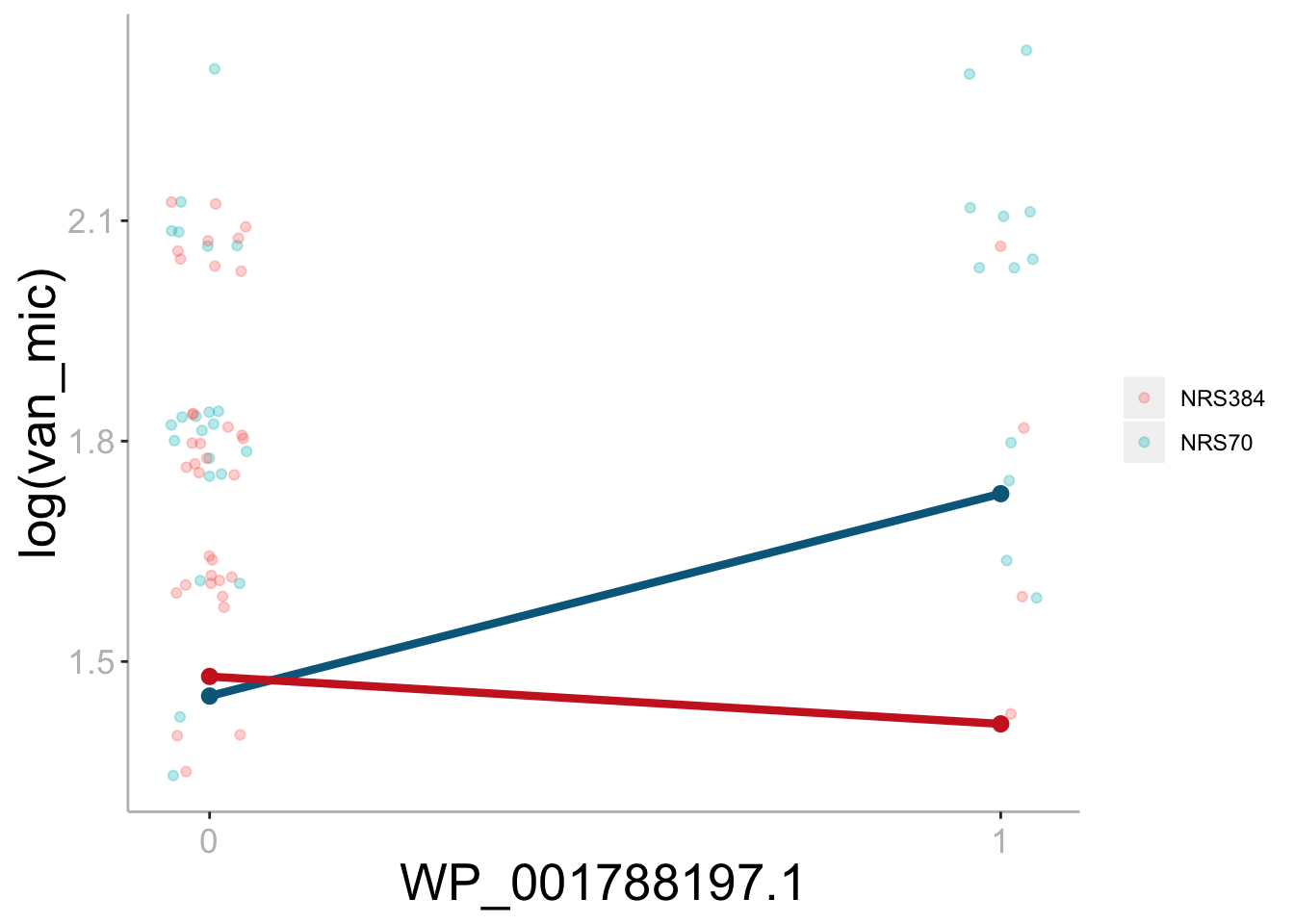

Supplement: Supplemental Information 2 — The difference in slope illustrates the interaction effect between rpoC and genetic background. The effect of rpoC on vancomycin MIC is 0.31 higher in NRS70 than in NRS384 (uncorrected p = 0.028). Bonferroni corrected significance threshold: ɑ = 0.00056. [file peerj-09-11764-s002.png]

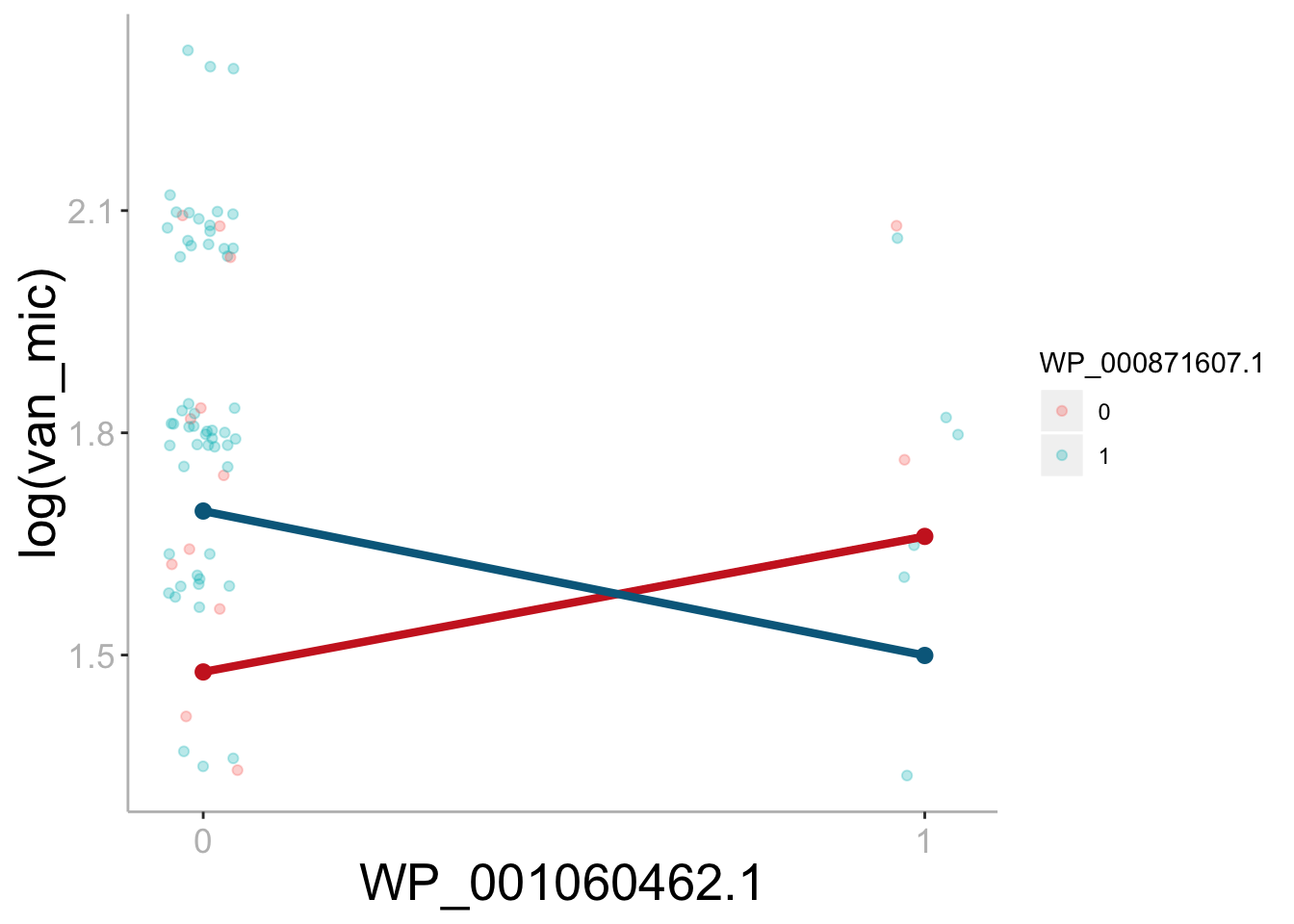

Supplement: Supplemental Information 3 — The difference in slope illustrates the interaction effect between sdrC and walK. The effect of sdrC.1 is 0.161 lower when a walK mutation is present (uncorrected p = 0.0472). Bonferroni corrected significance threshold: ɑ = 0.00056. [file peerj-09-11764-s003.png]
