# Supplementary material for: Effect of genetic background on the evolution of Vancomycin-Intermediate Staphylococcus aureus (VISA)
Source: PeerJ. 2021 Jul 13;9:e11764. doi: 10.7717/peerj.11764 (PMC8284308; doi:10.7717/peerj.11764)
Supplement: Supplemental Information 4 — Mutations were called with breseq. Daptomycin MICs were measured using E-test, and vancomycin MICs were measured using BMD. [file peerj-09-11764-s004.docx]

Strain Dapto MIC(μg/ml) Vanc MIC(μg/ml)

NRS70 0.38 1

NRS70.8.1 0.19 8

| **Type** | **Mutation** |
| --- | --- |
| Coding | SA_RS00245 A567V  SA_RS02930 R140H  SA_RS04395 S169R  SA_RS05165 I188T  SA_RS07265 G47G  SA_RS09765 A151V |
| Deletions | [SA_RS14675]-[SA_RS06275] bp 1258431-1258505 |

NRS70.8.2 3 6

| **Type** | **Mutation** |
| --- | --- |
| Coding | SA_RS02930 S143P  SA_RS08110 S125L  SA_RS09765 A151V  SA_RS11395 V87A |
| Intergenic | SA_RS09480 bp1916338 A |

NRS70.8.3 2 8

| **Type** | **Mutation** |
| --- | --- |
| Coding | SA_RS00245 L10F  SA_RS02640 R103K  SA_RS03550 K197N  SA_RS09760 S329L |
| Insertions | SA_RS07890 bp1602858 TTTTCC |

NRS70.8.4 3 10

| **Type** | **Mutation** |
| --- | --- |
| Coding | SA_RS00245 T367N  SA_RS02930 G489E  SA_RS09765 A151P  SA_RS11720 H33Y |

NRS70.8.5 1 6

| **Type** | **Mutation** |
| --- | --- |
| Coding | SA_RS00245 R86C  SA_RS09665 Y119D  SA_RS09755 D194N  SA_RS09760 K231I |
| Intergenic | SA_RS04580/SA_RS04585 bp908862 T |
| Insertions | SA_RS05350 bp1071102 A |

NRS70.8.6 3 10

| **Type** | **Mutation** |
| --- | --- |
| Coding | SA_RS00245 A567V  SA_RS02935 A729T  SA_RS09765 L57F  SA_RS09835 I48I |
| Insertions | SA_RS12325 bp2412690 A |

NRS70.8.7 3 8

| **Type** | **Mutation** |
| --- | --- |
| Coding | SA_RS00245 A243V  SA_RS02260 P184P  SA_RS02930 P1063S  SA_RS09665 H69R  SA_RS09765 V129E |

NRS70.8.8 2 8

| **Type** | **Mutation** |
| --- | --- |
| Coding | SA_RS00245 G223S  SA_RS00495 M647R  SA_RS02870 R146L  SA_RS02930 H481Y  SA_RS02930 G492D  SA_RS09765 S7L |
| Deletions | SA_RS07890 bp 1603260-1603260 |

NRS70.8.9 1.5 8

| **Type** | **Mutation** |
| --- | --- |
| Coding | SA_RS02935 F417I  SA_RS03025 A515V  SA_RS05350 Q234*  SA_RS08975 W248*  SA_RS09765 A152V  SA_RS10755 G124D  SA_RS11085 S513F  SA_RS11710 G14R |
| Intergenic | SA_RS09220/SA_RS09225 bp1873637 A SA_RS09630/SA_RS09635 bp1925100 A |
| Deletions | SA_RS05215 bp 1044309-1044311 |

NRS70.8.10 3 8

| **Type** | **Mutation** |
| --- | --- |
| Coding | SA_RS00245 L10F  SA_RS02930 P963S  SA_RS03415 T118I  SA_RS03545 T69I  SA_RS09765 P174Q |
| Deletions | [SA_RS07900]-[SA_RS07905] bp 1604326-1604373 |

NRS70.8.11 2 8

| **Type** | **Mutation** |
| --- | --- |
| Coding | SA_RS00245 V380I  SA_RS02935 T1119I  SA_RS08975 L106F  SA_RS09760 I32S  SA_RS11285 V78G |
| Intergenic | SA_RS13025/SA_RS14955 bp2544912 G |
| Insertions | SA_RS12105 bp2368936 A |
| Deletions | SA_RS06500 bp 1309898-1309898 |

NRS70.8.12 2 5

| **Type** | **Mutation** |
| --- | --- |
| Coding | SA_RS00245 T357I  SA_RS02930 P963T  SA_RS09765 A151P |
| Intergenic | SA_RS13210/SA_RS13220 bp2590880 T |
| Deletions | [SA_RS10065]-[SA_RS10405] bp 2005335-2049591 |

NRS70.8.13 3 10

| **Type** | **Mutation** |
| --- | --- |
| Coding | SA_RS00245 G223S  SA_RS02930 A862V  SA_RS02935 K474E  SA_RS09765 Y177S |
| Intergenic | SA_RS09370/SA_RS09375 bp1892676 T |
| Insertions | SA_RS06060 bp1209451 T |

NRS70.8.14 1.5 4

| **Type** | **Mutation** |
| --- | --- |
| Coding | SA_RS00245 A243V  SA_RS08315 L31F  SA_RS08430 A326E |
| Intergenic | SA_RS14715 bp1440134 C |
| Substitutions | SA_RS10010 bp1996415 GT |
| Insertions | SA_RS05375 bp1076333 A |
| Deletions | [SA_RS05345]-[SA_RS05350] bp 1070207-1071627 SA_RS09410 bp 1901568-1901848 |

NRS70.8.15 1 6

| **Type** | **Mutation** |
| --- | --- |
| Coding | SA_RS00245 R86C  SA_RS02930 R140H  SA_RS09765 V161F  SA_RS11560 Q376K |
| Deletions | [SA_RS10065]-[SA_RS10405] bp 2005335-2049591 |

NRS70.8.17 3 6

| **Type** | **Mutation** |
| --- | --- |
| Coding | SA_RS00245 R86C  SA_RS02930 R483H  SA_RS09765 A21P  SA_RS11655 P18S |
| Deletions | SA_RS09765 bp 1949024-1949024 |

NRS70.8.18 0.75 6

| **Type** | **Mutation** |
| --- | --- |
| Coding | SA_RS00245 E236D  SA_RS02930 R483H  SA_RS09765 P174S SA_RS12470 R76L |
| Intergenic | SA_RS09370/SA_RS09375 bp1892630 A |
| Insertions | SA_RS00225 bp23535 T |

NRS70.8.19 4 8

| **Type** | **Mutation** |
| --- | --- |
| Coding | SA_RS02930 A862E  SA_RS02935 L795F  SA_RS06100 Q183*  SA_RS09410 M1M  SA_RS09765 P174S |
| Deletions | SA_RS00245 bp 26758-26760 [SA_RS10065]-[SA_RS10405] bp 2005335-2049591 |

NRS70.8.20 3 5

| **Type** | **Mutation** |
| --- | --- |
| Coding | SA_RS02935 H449Q  SA_RS05975 V53L  SA_RS09765 T125I  SA_RS11720 G58R |
| Intergenic | SA_RS06245/SA_RS06250 bp1248770 T SA_RS11625/SA_RS11630 bp2294399 T |
| Deletions | SA_RS00245 bp 26758-26760 |

NRS70.8.23 2 8

| **Type** | **Mutation** |
| --- | --- |
| Coding | SA_RS00245 S9T  SA_RS02930 G491V  SA_RS09765 Y220C |
| Deletions | SA_RS01755 bp 360040-360040 |

NRS70.8.24 1.5 4

| **Type** | **Mutation** |
| --- | --- |
| Coding | SA_RS00240 I142N  SA_RS02930 P963S  SA_RS09765 S7L  SA_RS11975 L209L |

NRS70.8.25 1 5

| **Type** | **Mutation** |
| --- | --- |
| Coding | SA_RS00240 Q230*  SA_RS02935 G765V  SA_RS08935 A318T  SA_RS09760 I317F |

NRS70.8.26 3 8

| **Type** | **Mutation** |
| --- | --- |
| Coding | SA_RS00245 R86C  SA_RS02935 P431L  SA_RS09765 A152T |
| Intergenic | SA_RS11245/SA_RS11250 bp2212403 T |

NRS70.8.29 2 6

| **Type** | **Mutation** |
| --- | --- |
| Coding | SA_RS09765 A151V |
| Deletions | SA_RS00245 bp 26758-26760 |

NRS70.8.30 2 6

| **Type** | **Mutation** |
| --- | --- |
| Coding | SA_RS00245 A243V  SA_RS02640 P259L  SA_RS06000 F39L  SA_RS07270 C197Y  SA_RS09760 R122Q  SA_RS11285 V78G  SA_RS14000 M126V |

NRS70.8.31 3 8

| **Type** | **Mutation** |
| --- | --- |
| Coding | SA_RS02935 R958P  SA_RS06060 Y541D  SA_RS09375 Q263*  SA_RS09765 Y130C |
| Intergenic | SA_RS09510 bp1917044 G |

NRS70.8.32 2 6

| **Type** | **Mutation** |
| --- | --- |
| Coding | SA_RS00245 L433S  SA_RS00700 L167L  SA_RS00745 A139V  SA_RS01145 V270V  SA_RS01930 P40S  SA_RS03030 D1127D  SA_RS03030 D1129D  SA_RS03030 S1130S  SA_RS03030 D1135D  SA_RS03030 D1139D  SA_RS03030 S1250S  SA_RS03030 D1251D  SA_RS03030 D1253D  SA_RS03445 K85E  SA_RS04220 H91R  SA_RS04255 P60L  SA_RS06730 D837G  SA_RS07265 A212A  SA_RS07425 G239D  SA_RS07635 G120G  SA_RS09760 K336N  SA_RS09990 W25R  SA_RS14045 A42A |
| Intergenic | SA_RS05470/SA_RS05475 bp1097601 G  SA_RS13065 bp2554008 C |
| Deletions | SA_RS00300/SA_RS00305 bp 39864-39864  SA_RS05885 bp 1170286-1170286  SA_RS06430 bp 1294686-1294686  SA_RS11865 bp 2326495-2326495  SA_RS12535 bp 2456314-2456314 |

NRS70.8.33 1.5 6

| **Type** | **Mutation** |
| --- | --- |
| Coding | SA_RS00245 R86C  SA_RS07270 C197Y  SA_RS07860 P266L  SA_RS09765 P174S |

NRS70.8.34 2 6

| **Type** | **Mutation** |
| --- | --- |
| Coding | SA_RS00245 G223S  SA_RS02530 H183Y  SA_RS02930 A620E  SA_RS04865 Q535H  SA_RS09765 G140R |
| Intergenic | SA_RS12745/SA_RS12750 bp2492262 A |
| Deletions | [SA_RS04005] bp 803223-803378 [SA_RS10065]-[SA_RS10405] bp 2005335-2049591 |

NRS70.8.35 2 5

| **Type** | **Mutation** |
| --- | --- |
| Coding | SA_RS01750 G5G  SA_RS06415 P84L  SA_RS09410 Q30*  SA_RS09765 A59E |
| Deletions | SA_RS00245 bp 26758-26760 |

NRS70.8.36 3 6

| **Type** | **Mutation** |
| --- | --- |
| Coding | SA_RS00245 R86C  SA_RS07270 C197Y  SA_RS07860 P266L  SA_RS09765 P174S |

NRS70.8.37 2 6

| **Type** | **Mutation** |
| --- | --- |
| Coding | SA_RS00245 A567V  SA_RS02930 A1085V  SA_RS04950 T5P  SA_RS09765 P174S  SA_RS09865 A438T |
| Deletions | SA_RS05050/SA_RS05055 bp 1011765-1011765 SA_RS11090/SA_RS11095 bp 2180285-2180285 |

NRS70.8.38 1.5 8

| **Type** | **Mutation** |
| --- | --- |
| Coding | SA_RS00240 P216H  SA_RS02935 P482R  SA_RS07270 C197Y  SA_RS09765 A152T |
| Intergenic | SA_RS14715 bp1440134 C |

NRS70.8.39 2 6

| **Type** | **Mutation** |
| --- | --- |
| Coding | SA_RS00240 P216H  SA_RS02935 A778V  SA_RS09765 P126L |

NRS70.8.40 3 6

| **Type** | **Mutation** |
| --- | --- |
| Coding | SA_RS00245 R86C  SA_RS02935 D485N  SA_RS09765 P126L |
| Intergenic | SA_RS05420/SA_RS05425 bp1085725 A |
| Deletions | SA_RS04005 bp 802713-802796 |

NRS384 0.38 1

NRS384.8.1 1.5 5

| **Type** | **Mutation** |
| --- | --- |
| Coding | NRS_23 D522G  NRS_30 G358S  NRS_251 M137I  NRS_528 R428H  NRS_528 P458R  NRS_553 L46F  NRS_1170 M103I  NRS_1525 A65T  NRS_1606 V350V  NRS_1726 R63R  NRS_1899 L57F  NRS_2015 R449C  NRS_2289 V125I  NRS_2450 A508V  NRS_2533 A625T  NRS_2572 R260C  NRS_2604 T122M |
| Intergenic | NRS_30 I185T  NRS_765 T214I  NRS_1568 R231C  NRS_1705 K84E  NRS_1899 P126L |
| Substitutions | NRS_158/NRS_161 bp169212 GA NRS_2521/NRS_2522 bp2680094 GT NRS_2521/NRS_2522 bp2680102 G  NRS_2521/NRS_2522 bp2680108 CC NRS_2521/NRS_2522 bp2680117 TGT  NRS_2578 bp2741433 TGCGAGACTGAACTCCAT |
| Insertions | NRS_2521/NRS_2522 bp2680071 TCAG NRS_2521/NRS_2522 bp2680086 A NRS_2521/NRS_2522 bp2680134 T |
| Deletions | NRS_1619 bp 1741462-1741492 NRS_1842/NRS_1843 bp 1995021-1995072 |

NRS384.8.2 4 6

| **Type** | **Mutation** |
| --- | --- |
| Coding | NRS_30 M291I  NRS_103 G54G  NRS_666 L168I  NRS_1899 L57F |
| Substitutions | NRS_158/NRS_161 bp169212 GA  NRS_772/NRS_773 bp841847 CG NRS_921/NRS_924 bp991581 GCG  NRS_2521/NRS_2522 bp2680094 GT NRS_2521/NRS_2522 bp2680102 G NRS_2521/NRS_2522 bp2680108 CC  NRS_2521/NRS_2522 bp2680117 TGT  NRS_2578 bp2741433 TGCGAGACTGAACTCCAT |
| Insertions | NRS_1595 bp1716766 GG  NRS_2521/NRS_2522 bp2680071 TCAG NRS_2521/NRS_2522 bp2680086 A NRS_2521/NRS_2522 bp2680134 T |

NRS384.8.3 2 5

| **Type** | **Mutation** |
| --- | --- |
| Coding | NRS_30 Q216E  NRS_491 G217V  NRS_688 M76T  NRS_1887 R103* |
| Substitutions | NRS_2521/NRS_2522 bp2680094 GT NRS_2521/NRS_2522 bp2680102 G  NRS_2521/NRS_2522 bp2680108 CC NRS_2521/NRS_2522 bp2680117 TGT  NRS_2578 bp2741433 TGCGAGACTGAACTCCAT |
| Insertions | NRS_1897 bp2025818 TACGGTTACGCATTTTCA NRS_2521/NRS_2522 bp2680071 TCAG NRS_2521/NRS_2522 bp2680086 A NRS_2521/NRS_2522 bp2680134 T |
| Deletions | NRS_1951-NRS_2005 bp 2084668-2127716 |

NRS384.8.4 1.5 6

| **Type** | **Mutation** |
| --- | --- |
| Coding | NRS_30 G223D  NRS_262 T47A  NRS_666 R308*  NRS_1019 P356S  NRS_1899 P126L |
| Substitutions | NRS_158/NRS_161 bp169212 GA  NRS_158/NRS_161 bp169317 AG  NRS_772/NRS_773 bp841847 CG NRS_2521/NRS_2522 bp2680094 GT NRS_2521/NRS_2522 bp2680102 G NRS_2521/NRS_2522 bp2680108 CC  NRS_2521/NRS_2522 bp2680117 TGT  NRS_2578 bp2741433 TGCGAGACTGAACTCCAT |
| Insertions | NRS_2521/NRS_2522 bp2680071 TCAG NRS_2521/NRS_2522 bp2680086 A NRS_2521/NRS_2522 bp2680134 T |
| Deletions | NRS_1842/NRS_1843 bp 1995021-1995072 |

NRS384.8.5 2 8

| **Type** | **Mutation** |
| --- | --- |
| Coding | NRS_30 G223D  NRS_1319 G14V  NRS_1352 G8624A  NRS_1899 A152T |
| Substitutions | NRS_158/NRS_161 bp169212 GA NRS_921/NRS_924 bp991581 GCGT NRS_2521/NRS_2522 bp2680094 GT NRS_2521/NRS_2522 bp2680102 G NRS_2521/NRS_2522 bp2680108 CC NRS_2521/NRS_2522 bp2680117 TGT  NRS_2578 bp2741433 TGCGAGACTGAACTCCAT |
| Insertions | NRS_831/NRS_832 bp892268 A NRS_2521/NRS_2522 bp2680071 TCAG NRS_2521/NRS_2522 bp2680086 A NRS_2521/NRS_2522 bp2680134 T |
| Deletions | NRS_1842/NRS_1843 bp 1995021-1995072 |

NRS384.8.6 0.125 8

| **Type** | **Mutation** |
| --- | --- |
| Coding | NRS_30 R204G  NRS_644 S116G  NRS_661 N71Y  NRS_1898 G318S  NRS_1898 S196T  NRS_2149 Q95L  NRS_2276 V139I |
| Substitutions | NRS_772/NRS_773 bp841847 CG NRS_2521/NRS_2522 bp2680094 GT NRS_2521/NRS_2522 bp2680102 G  NRS_2521/NRS_2522 bp2680108 CC NRS_2521/NRS_2522 bp2680117 TGT  NRS_2578 bp2741433 TGCGAGACTGAACTCCAT |
| Insertions | NRS_2521/NRS_2522 bp2680071 TCAG NRS_2521/NRS_2522 bp2680086 A NRS_2521/NRS_2522 bp2680134 T |
| Deletions | NRS_1842/NRS_1843 bp 1995021-1995072 |

NRS384.8.7 0.25 8

| **Type** | **Mutation** |
| --- | --- |
| Coding | NRS_30 G223C  NRS_591 G52G  NRS_1085 P266T  NRS_1898 T331I |
| Substitutions | NRS_921/NRS_924 bp991579 TAGCGT NRS_921/NRS_924 bp991586 GTGTATTCAGA NRS_2521/NRS_2522 bp2680094 GT  NRS_2521/NRS_2522 bp2680102 G NRS_2521/NRS_2522 bp2680108 CC NRS_2521/NRS_2522 bp2680117 TGT  NRS_2578 bp2741433 TGCGAGACTGAACTCCAT |
| Insertions | NRS_2521/NRS_2522 bp2680071 TCAG NRS_2521/NRS_2522 bp2680086 A NRS_2521/NRS_2522 bp2680134 T |
| Deletions | NRS_1650 bp 1775202-1775243  NRS_2214 bp 2358024-2358039  NRS_2649 bp 2837771-2837772 |

NRS384.8.8 2 6

| **Type** | **Mutation** |
| --- | --- |
| Coding | NRS_30 T217K  NRS_924 T219M  NRS_1019 G416D  NRS_1899 P174Q  NRS_1899 V36F |
| Substitutions | NRS_2521/NRS_2522 bp2680094 GT NRS_2521/NRS_2522 bp2680102 G NRS_2521/NRS_2522 bp2680108 CC NRS_2521/NRS_2522 bp2680117 TGT  NRS_2578 bp2741433 TGCGAGACTGAACTCCAT |
| Insertions | NRS_2521/NRS_2522 bp2680071 TCAG NRS_2521/NRS_2522 bp2680086 A  NRS_2521/NRS_2522 bp2680134 T |

NRS384.8.9 6 8

| **Type** | **Mutation** |
| --- | --- |
| Coding | NRS_30 V196F  NRS_1726 R63R |
| Substitutions | NRS_158/NRS_161 bp169212 GA  NRS_2578 bp2741433 TGCGAGACTGAACTCCAT |

NRS384.8.10 4 8

| **Type** | **Mutation** |
| --- | --- |
| Coding | NRS_30 R286S  NRS_110 S183S  NRS_110 K185Q  NRS_761 G30R  NRS_1899 D134G |
| Substitutions | NRS_772/NRS_773 bp841847 CG  NRS_2521/NRS_2522 bp2679940 CG  NRS_2521/NRS_2522 bp2679946 CG NRS_2521/NRS_2522 bp2679952 AG NRS_2521/NRS_2522 bp2680094 GT NRS_2521/NRS_2522 bp2680102 G  NRS_2521/NRS_2522 bp2680108 CC NRS_2521/NRS_2522 bp2680117 TGT  NRS_2578 bp2741433 TGCGAGACTGAACTCCAT |

NRS384.8.11 1.5 6

| **Type** | **Mutation** |
| --- | --- |
| Coding | NRS_30 T270N  NRS_1563 M1M  NRS_1899 P174A |
| Substitutions | NRS_158/NRS_161 bp169212 GA  NRS_772/NRS_773 bp841847 CG  NRS_2521/NRS_2522 bp2680094 GT NRS_2521/NRS_2522 bp2680102 G NRS_2521/NRS_2522 bp2680108 CC NRS_2521/NRS_2522 bp2680117 TGT  NRS_2578 bp2741433 TGCGAGACTGAACTCCAT |
| Insertions | NRS_2521/NRS_2522 bp2680071 TCAG NRS_2521/NRS_2522 bp2680086 A  NRS_2521/NRS_2522 bp2680134 T |
| Deletions | NRS_1142 bp 1225371-1225372  NRS_1842/NRS_1843 bp 1995021-1995072 |

NRS384.8.12 0.38 8

| **Type** | **Mutation** |
| --- | --- |
| Coding | NRS_9 A136T  NRS_30 V196F  NRS_640 Q182K  NRS_700 A268E  NRS_1899 A59E |
| Substitutions | NRS_158/NRS_161 bp169212 GA NRS_921/NRS_924 bp991579 TAGCGT NRS_921/NRS_924 bp991586 GTGTATTCAGA  NRS_2521/NRS_2522 bp2680094 GT NRS_2521/NRS_2522 bp2680102 G NRS_2521/NRS_2522 bp2680108 CC  NRS_2521/NRS_2522 bp2680117 TGT  NRS_2578 bp2741433 TGCGAGACTGAACTCCAT |
| Insertions | NRS_2521/NRS_2522 bp2680071 TCAG NRS_2521/NRS_2522 bp2680086 A  NRS_2521/NRS_2522 bp2680134 T |

NRS384.8.13 2 8

| **Type** | **Mutation** |
| --- | --- |
| Coding | NRS_30 A189D  NRS_540 R140H  NRS_871 A320V  NRS_1352 T2109I  NRS_1898 I100R  NRS_2243 L344L |
| Substitutions | NRS_158/NRS_161 bp169212 GA  NRS_511 bp556345 AT  NRS_921/NRS_924 bp991579 TAGCGT |
| Insertions | NRS_1779/NRS_1781 bp1929108 T  NRS_2521/NRS_2522 bp2680071 TCAG NRS_2521/NRS_2522 bp2680086 A NRS_2521/NRS_2522 bp2680134 T |
| Deletions | NRS_1352 bp 1485612-1485613  NRS_1779/NRS_1781 bp 1929087-1929089 NRS_1842/NRS_1843 bp 1995021-1995072 |

NRS384.8.14 3 6

| **Type** | **Mutation** |
| --- | --- |
| Coding | NRS_30 A189D  NRS_664 A576V  NRS_700 G254D  NRS_1822 V9D  NRS_1898 Q289K |
| Substitutions | NRS_158/NRS_161 bp169212 GA  NRS_158/NRS_161 bp169317 AG  NRS_158/NRS_161 bp169334 GC NRS_2521/NRS_2522 bp2680094 GT NRS_2521/NRS_2522 bp2680102 G NRS_2521/NRS_2522 bp2680108 CC  NRS_2521/NRS_2522 bp2680117 TGT  NRS_2578 bp2741433 TGCGAGACTGAACTCCAT |
| Insertions | NRS_2521/NRS_2522 bp2680071 TCAG NRS_2521/NRS_2522 bp2680086 A  NRS_2521/NRS_2522 bp2680134 T |

NRS384.8.15 3 6

| **Type** | **Mutation** |
| --- | --- |
| Coding | NRS_30 G223D  NRS_110 K185Q  NRS_541 T775I  NRS_544 R81L  NRS_559 D775D  NRS_1726 R63R  NRS_1898 G318V |
| Substitutions | NRS_2578 bp2741433 TGCGAGACTGAACTCCAT |
| Deletions | NRS_1842/NRS_1843 bp 1995021-1995072 |

NRS384.8.16 1.5 4

| **Type** | **Mutation** |
| --- | --- |
| Coding | NRS_30 G223D  NRS_541 T775I  NRS_544 R81L  NRS_559 S770S  NRS_559 D775D  NRS_1726 R63R  NRS_1898 G318V |
| Substitutions | NRS_158/NRS_161 bp169212 GA NRS_921/NRS_924 bp991579 TAGCGT NRS_921/NRS_924 bp991586 GTGTATTCAGA NRS_2578 bp2741433 TGCGAGACTGAACTCCAT |
| Insertions | NRS_1939 bp2074104 ACTAAT |
| Deletions | NRS_1842/NRS_1843 bp 1995021-1995072 |

NRS384.8.17 1.5 5

| **Type** | **Mutation** |
| --- | --- |
| Coding | NRS_30 V15L  NRS_750 A66E  NRS_1899 G140E |
| Substitutions | NRS_158/NRS_161 bp169212 GA NRS_921/NRS_924 bp991586 GTGTATTCAGA NRS_2521/NRS_2522 bp2680094 GT NRS_2521/NRS_2522 bp2680102 G NRS_2521/NRS_2522 bp2680108 CC NRS_2521/NRS_2522 bp2680117 TGT  NRS_2578 bp2741433 TGCGAGACTGAACTCCAT |
| Insertions | NRS_2521/NRS_2522 bp2680071 TCAG NRS_2521/NRS_2522 bp2680086 A NRS_2521/NRS_2522 bp2680134 T |

NRS384.8.18 2 5

| **Type** | **Mutation** |
| --- | --- |
| Coding | NRS_30 A567D  NRS_540 A477V  NRS_540 A862V  NRS_640 N43T  NRS_1898 R332H |
| Substitutions | NRS_158/NRS_161 bp169212 GA  NRS_158/NRS_161 bp169317 AG  NRS_772/NRS_773 bp841847 CG  NRS_921/NRS_924 bp991579 TAGCGT NRS_921/NRS_924 bp991586 GTGTATTCAGA NRS_2521/NRS_2522 bp2679946 CG NRS_2521/NRS_2522 bp2679952 AG NRS_2521/NRS_2522 bp2680094 GT NRS_2521/NRS_2522 bp2680102 G NRS_2521/NRS_2522 bp2680108 CC NRS_2521/NRS_2522 bp2680117 TGT  NRS_2578 bp2741433 TGCGAGACTGAACTCCAT |
| Insertions | NRS_2521/NRS_2522 bp2680071 TCAG NRS_2521/NRS_2522 bp2680086 A NRS_2521/NRS_2522 bp2680134 T |
| Deletions | NRS_1842/NRS_1843 bp 1995021-1995072 |

NRS384.8.19 2 6

| **Type** | **Mutation** |
| --- | --- |
| Coding | NRS_540 T622I  NRS_559 S770S  NRS_559 D775D  NRS_766 S69P  NRS_979 A71V  NRS_1278 D606G  NRS_1726 R63R  NRS_1899 G56D  NRS_2189 Q98K |
| Substitutions | NRS_158/NRS_161 bp169212 GA  NRS_158/NRS_161 bp169317 AG  NRS_158/NRS_161 bp169334 GC  NRS_772/NRS_773 bp841847 CG NRS_921/NRS_924 bp991579 TAGCGT NRS_921/NRS_924 bp991586 GTGTATTCAGA NRS_2521/NRS_2522 bp2680094 GT NRS_2521/NRS_2522 bp2680102 G NRS_2521/NRS_2522 bp2680108 CC NRS_2521/NRS_2522 bp2680117 TGT  NRS_2578 bp2741433 TGCGAGACTGAACTCCAT |
| Insertions | NRS_1202 bp1298123 AAAGAA  NRS_2521/NRS_2522 bp2680071 TCAG NRS_2521/NRS_2522 bp2680086 A  NRS_2521/NRS_2522 bp2680134 T |
| Deletions | NRS_1821/NRS_1822 bp 1971513-1971514 NRS_1842/NRS_1843 bp 1995021-1995072 |

NRS384.8.20 3 6

| **Type** | **Mutation** |
| --- | --- |
| Coding | NRS_30 I185T  NRS_765 T214I  NRS_1568 R231C  NRS_1705 K84E  NRS_1899 P126L |
| Substitutions | NRS_158/NRS_161 bp169212 GA  NRS_158/NRS_161 bp169317 AG  NRS_921/NRS_924 bp991586 GTGTATTCAGA NRS_2521/NRS_2522 bp2680094 GT NRS_2521/NRS_2522 bp2680102 G  NRS_2521/NRS_2522 bp2680108 CC NRS_2521/NRS_2522 bp2680117 TGT  NRS_2578 bp2741433 TGCGAGACTGAACTCCAT |
| Insertions | NRS_2521/NRS_2522 bp2680071 TCAG NRS_2521/NRS_2522 bp2680086 A NRS_2521/NRS_2522 bp2680134 T |
| Deletions | NRS_2340 bp 2475617-2475618 |

NRS384.8.21 3 5

| **Type** | **Mutation** |
| --- | --- |
| Coding | NRS_30 G358S  NRS_37 S213S  NRS_541 A371P  NRS_1898 I333F |
| Substitutions | NRS_1779/NRS_1781 bp1929111 TT  NRS_1779/NRS_1781 bp1929117 AA |
| Insertions | NRS_261 bp301825 CCTGAAGATAAGTACTTA |
| Deletions | NRS_158/NRS_161 bp 169257-169258 NRS_1868/NRS_1869 bp 1997576-1997577 |

NRS384.8.22 3 5

| **Type** | **Mutation** |
| --- | --- |
| Coding | NRS_30 Q370K  NRS_37 S213S  NRS_750 S95N  NRS_1899 G56S  NRS_2625 Q204* |
| Substitutions | NRS_2521/NRS_2522 bp2679946 CG  NRS_2521/NRS_2522 bp2679952 AG |
| Deletions | NRS_1868/NRS_1869 bp 1997576-1997577  NRS_2620 bp 2799855-2799867 |

NRS384.8.23 4 5

| **Type** | **Mutation** |
| --- | --- |
| Coding | NRS_30 G223D  NRS_37 S213S  NRS_640 G36D  NRS_662 T199I  NRS_1899 S7L |
| Insertions | NRS_98/NRS_99 bp97236 T |
| Deletions | NRS_158/NRS_161 bp 169257-169258 NRS_1868/NRS_1869 bp 1997576-1997577 |

NRS384.8.24 2 5

| **Type** | **Mutation** |
| --- | --- |
| Coding | NRS_30 G358S  NRS_37 S213S  NRS_161 D761A  NRS_540 A862V  NRS_559 S770S  NRS_559 D775D  NRS_1197 G140D  NRS_1551 C194Y  NRS_1726 R63R  NRS_1898 V236G |
| Substitutions | NRS_501 bp546823 TC NRS_1779/NRS_1781 bp1929111 TT  NRS_1779/NRS_1781 bp1929117 AA |
| Deletions | NRS_158/NRS_161 bp 169257-169258 |

NRS384.8.25 3 6

| **Type** | **Mutation** |
| --- | --- |
| Coding | NRS_30 M291I  NRS_37 S213S  NRS_1122 S26C  NRS_1595 E50G  NRS_1898 S167I |
| Substitutions | NRS_1779/NRS_1781 bp1929111 TT  NRS_1779/NRS_1781 bp1929117 AA NRS_2521/NRS_2522 bp2679946 CG NRS_2521/NRS_2522 bp2679952 AG |
| Insertions | NRS_700 bp758712 TG |
| Deletions | NRS_158/NRS_161 bp 169257-169258 NRS_1779/NRS_1781 bp 1929087-1929089 NRS_1868/NRS_1869 bp 1997576-1997577 |

NRS384.8.26 3 8

| **Type** | **Mutation** |
| --- | --- |
| Coding | NRS_30 A243T  NRS_37 S213S  NRS_258 H36Q  NRS_1899 V129G  NRS_2116 F507C |
| Deletions | NRS_158/NRS_161 bp 169257-169258 NRS_1868/NRS_1869 bp 1997576-1997577 |

NRS384.8.27 1.5 5

| **Type** | **Mutation** |
| --- | --- |
| Coding | NRS_30 G223D  NRS_37 S213S  NRS_662 V111D  NRS_1897 A113D  NRS_1898 Q126K |
| Insertions | NRS_1016 bp1089410 A |
| Deletions | NRS_158/NRS_161 bp 169257-169258 |

NRS384.8.29 3 8

| **Type** | **Mutation** |
| --- | --- |
| Coding | NRS_30 M220I  NRS_37 S213S  NRS_541 G413C  NRS_559 S770S  NRS_559 D775D  NRS_759 A714V  NRS_904 N202Y  NRS_985 A267T  NRS_1018 A389V  NRS_1136 V659V  NRS_1182 V151I  NRS_1319 G16C  NRS_1436 G28R  NRS_1726 R63R  NRS_1898 D26Y  NRS_2042 S38F |
| Substitutions | NRS_2521/NRS_2522 bp2679946 CG  NRS_2521/NRS_2522 bp2679952 AG |

NRS384.8.30 1.5 6

| **Type** | **Mutation** |
| --- | --- |
| Coding | NRS_30 G223C  NRS_37 S213S  NRS_559 S770S  NRS_559 D775D  NRS_1726 R63R  NRS_1899 G140E  NRS_2231 Y46N |
| Substitutions | NRS_2521/NRS_2522 bp2679946 CG  NRS_2521/NRS_2522 bp2679952 AG |
| Deletions | NRS_158/NRS_161 bp 169257-169258 NRS_1868/NRS_1869 bp 1997576-1997577 |

NRS384.8.31 1.5 4

| **Type** | **Mutation** |
| --- | --- |
| Coding | NRS_30 R263L  NRS_37 S213S  NRS_1899 P126L |
| Deletions | NRS_158/NRS_161 bp 169257-169258  NRS_1469 bp 1597224-1597229 NRS_1868/NRS_1869 bp 1997576-1997577  NRS_2364 bp 2501671-2501672 |

NRS384.8.32 2 6

| **Type** | **Mutation** |
| --- | --- |
| Coding | NRS_30 G358S  NRS_662 W158C  NRS_665 P70L  NRS_1899 V199G |
| Substitutions | NRS_1779/NRS_1781 bp1929111 TT  NRS_1779/NRS_1781 bp1929117 AA |
| Deletions | NRS_158/NRS_161 bp 169257-169258 NRS_1779/NRS_1781 bp 1929087-1929089 NRS_1868/NRS_1869 bp 1997576-1997577 |

NRS384.8.33 2 8

| **Type** | **Mutation** |
| --- | --- |
| Coding | NRS_30 G223D  NRS_540 A477V  NRS_1550 E155D  NRS_1899 L57F |
| Substitutions | NRS_921/NRS_924 bp991579 TAGCGT |
| Deletions | NRS_158/NRS_161 bp 169257-169258 |

NRS384.8.34 1 5

| **Type** | **Mutation** |
| --- | --- |
| Coding | NRS_30 T188A  NRS_37 S213S  NRS_559 S770S  NRS_559 D775D  NRS_1726 R63R  NRS_1898 N291K |
| Deletions | NRS_158/NRS_161 bp 169257-169258 NRS_1868/NRS_1869 bp 1997576-1997577  NRS_2117 bp 2245138-2245264 |

NRS384.8.35 3 5

| **Type** | **Mutation** |
| --- | --- |
| Coding | NRS_30 G223S  NRS_37 S213S  NRS_540 G167S  NRS_640 Q182P  NRS_1899 Y220C |
| Substitutions | NRS_1779/NRS_1781 bp1929111 TT  NRS_1779/NRS_1781 bp1929117 AA NRS_2521/NRS_2522 bp2679952 AG |
| Deletions | NRS_158/NRS_161 bp 169257-169258  NRS_911 bp 980607-980608 NRS_1868/NRS_1869 bp 1997576-1997577 |

NRS384.8.37 0.75 4

| **Type** | **Mutation** |
| --- | --- |
| Coding | NRS_30 M426I  NRS_37 S213S  NRS_664 S295L  NRS_1899 N201Y |
| Substitutions | NRS_921/NRS_924 bp991579 TAGCGT |
| Deletions | NRS_158/NRS_161 bp 169257-169258 NRS_1779/NRS_1781 bp 1929087-1929088 NRS_1868/NRS_1869 bp 1997576-1997577 |

NRS384.8.38 1.5 4

| **Type** | **Mutation** |
| --- | --- |
| Coding | NRS_37 S213S  NRS_1899 P126L  NRS_2321 F143L |
| Substitutions | NRS_772/NRS_773 bp841847 CG |
| Deletions | NRS_32 bp 29528-29529  NRS_158/NRS_161 bp 169257-169258 NRS_1868/NRS_1869 bp 1997576-1997577 |

NRS384.8.39 3 6

| **Type** | **Mutation** |
| --- | --- |
| Coding | NRS_30 F184Y  NRS_37 S213S  NRS_540 R428S  NRS_1898 Q289R |
| Substitutions | NRS_1779/NRS_1781 bp1929111 TT  NRS_1779/NRS_1781 bp1929117 AA |
| Deletions | NRS_158/NRS_161 bp 169257-169258 NRS_1779/NRS_1781 bp 1929087-1929089 NRS_1868/NRS_1869 bp 1997576-1997577 |

NRS384.8.40 2 6

| **Type** | **Mutation** |
| --- | --- |
| Coding | NRS_30 G223D  NRS_1898 T331I  NRS_2215 D112G  NRS_2525 R394R |
| Deletions | NRS_158/NRS_161 bp 169257-169258 |

NRS123.8.1

| **Type** | **Mutation** |
| --- | --- |
| Coding | MW_RS00100 P13T  MW_RS00100 E63E  MW_RS00105 T195K  MW_RS05255 D400N  MW_RS09935 N291K |
| Intergenic | MW_RS03260/MW_RS03265 bp681212 T |

NRS123.8.2

| **Type** | **Mutation** |
| --- | --- |
| Coding | MW_RS00105 L10F  MW_RS02745 P1063S  MW_RS03390 K84N  MW_RS05245 Y92*  MW_RS08755 S105L  MW_RS09880 Q240* |

NRS123.8.4

| **Type** | **Mutation** |
| --- | --- |
| Coding | MW_RS00105 G223D  MW_RS05260 G45V  MW_RS09840 E156K  MW_RS09935 I82S |
| Substitutions | MW_RS09320/MW_RS09325 bp1894015 CATTACTTTGCTAATC |
| Deletions | MW_RS05925 bp 1203835-1203879 |

NRS123.8.5

| **Type** | **Mutation** |
| --- | --- |
| Coding | MW_RS00105 A243T  MW_RS01220 G166A  MW_RS02745 A1082V  MW_RS03165 L196* |

NRS123.8.6

| **Type** | **Mutation** |
| --- | --- |
| Coding | MW_RS00105 G223C  MW_RS06700 T345I  MW_RS09940 P126L |
| Substitutions | MW_RS09320/MW_RS09325 bp1894015 CATTACTTTGCTAATC |
| Insertions | MW_RS05245 bp1072848 TA |

NRS123.8.7

| **Type** | **Mutation** |
| --- | --- |
| Coding | MW_RS00105 G223C  MW_RS03390 S55L  MW_RS05255 W292*  MW_RS05920 G169D  MW_RS13740 V186I |
| Insertions | MW_RS09880 bp1979458 A |
| Deletions | [MW_RS10245]-[MW_RS10565] bp 2046219-2088820 |

NRS123.8.8

| **Type** | **Mutation** |
| --- | --- |
| Coding | MW_RS09170 A206V |

NRS123.8.9

| **Type** | **Mutation** |
| --- | --- |
| Coding | MW_RS00105 K262E  MW_RS02750 R958C  MW_RS03465 W153*  MW_RS14590 C26F  MW_RS07835 I291T  MW_RS09940 G140E  MW_RS11240 D120Y |
| Substitutions | MW_RS02765 bp578492 GT |

NRS123.8.10

| **Type** | **Mutation** |
| --- | --- |
| Coding | MW_RS00105 A292P  MW_RS02750 V800L  MW_RS09940 P126L |

NRS123.8.12

| **Type** | **Mutation** |
| --- | --- |
| Coding | MW_RS00105 R511P  MW_RS09940 A151V |
| Intergenic | MW_RS06140/MW_RS06145 bp1248897 C MW_RS08160/MW_RS08165 bp1654589 T |

NRS123.8.13

| **Type** | **Mutation** |
| --- | --- |
| Coding | MW_RS02750 N735K  MW_RS05245 Q132*  MW_RS09940 L49P |
| Intergenic | MW_RS09685 bp1957557 G |
| Insertions | MW_RS03870 bp798622 A |

NRS123.8.14

| **Type** | **Mutation** |
| --- | --- |
| Coding | MW_RS00105 G223D  MW_RS03330 T81I  MW_RS09940 T223K |
| Substitutions | MW_RS00735 bp162227 AT |

NRS123.8.15

| **Type** | **Mutation** |
| --- | --- |
| Coding | MW_RS00105 G223D  MW_RS01050 A93V  MW_RS09940 G140R |
| Deletions | [MW_RS10245]-[MW_RS10565] bp 2046219-2088820 |

NRS123.8.16

| **Type** | **Mutation** |
| --- | --- |
| Coding | MW_RS00105 M426I  MW_RS09940 P126L |
| Deletions | MW_RS03860 bp 797038-797049 MW_RS08165-[MW_RS08175] bp 1654517-1656936 MW_RS12340 bp 2424259-2424259 |

NRS123.8.17

| **Type** | **Mutation** |
| --- | --- |
| Coding | MW_RS00105 I29M  MW_RS02750 A803P  MW_RS08245 I191T |
| Intergenic | MW_RS09665 bp1957192 A |

NRS123.8.18

| **Type** | **Mutation** |
| --- | --- |
| Coding | MW_RS02750 R797H MW_RS09940 Q136P |
| Deletions | MW_RS12225/MW_RS12230 bp 2402868-2403002 |

NRS123.8.19

| **Type** | **Mutation** |
| --- | --- |
| Coding | MW_RS00105 S9T  MW_RS02745 A477V  MW_RS09935 S329L  MW_RS12765 L119* |

NRS123.8.20

| **Type** | **Mutation** |
| --- | --- |
| Coding | MW_RS00105 E373A  MW_RS02440 I13N  MW_RS08565 Y56N  MW_RS09940 T125I |
| Substitutions | MW_RS09320/MW_RS09325 bp1894019 CTT |
| Insertions | MW_RS09320/MW_RS09325 bp1894017 T |

NRS123.8.21

| **Type** | **Mutation** |
| --- | --- |
| Coding | MW_RS00105 L429R  MW_RS01070 P629H  MW_RS08130 A83E |
| Deletions | MW_RS14510-MW_RS02920 bp 617259-621100 MW_RS09940 bp 1989589-1989589 [MW_RS10245]-[MW_RS10565] bp 2046219-2088820 MW_RS13185/MW_RS13190 bp 2594611-2594611 |

NRS123.8.22

| **Type** | **Mutation** |
| --- | --- |
| Coding | MW_RS00105 G358S  MW_RS05790 A764S  MW_RS09935 V66G |
| Intergenic | MW_RS08160/MW_RS08165 bp1654588 T |

NRS123.8.24

| **Type** | **Mutation** |
| --- | --- |
| Coding | MW_RS00070 V632V  MW_RS02750 V800L  MW_RS03860 A233V  MW_RS09940 P126L |
| Intergenic | MW_RS09685 bp1957557 G |

NRS123.8.25

| **Type** | **Mutation** |
| --- | --- |
| Coding | MW_RS00105 G358S  MW_RS08615 Y298N  MW_RS09940 A152V |
| Intergenic | MW_RS08160/MW_RS08165 bp1654499 T |

NRS123.8.26

| **Type** | **Mutation** |
| --- | --- |
| Coding | MW_RS00105 G223D  MW_RS09935 S167I  MW_RS11175 W81* |
| Substitutions | MW_RS09320/MW_RS09325 bp1894019 CTT |
| Insertions | MW_RS09320/MW_RS09325 bp1894017 T |

NRS123.8.28

| **Type** | **Mutation** |
| --- | --- |
| Coding | MW_RS00105 D440Y  MW_RS02750 R779C  MW_RS10600 A75A |
| Intergenic | MW_RS00460/MW_RS00465 bp105695 C MW_RS04530/MW_RS04535 bp921644 T MW_RS07740/MW_RS07745 bp1580635 T |
| Substitutions | MW_RS09320/MW_RS09325 bp1894019 CTT |
| Insertions | MW_RS09320/MW_RS09325 bp1894017 T |

NRS123.8.29

| **Type** | **Mutation** |
| --- | --- |
| Coding | MW_RS00105 G358S  MW_RS02745 R406H  MW_RS09935 D290E |
| Substitutions | MW_RS09320/MW_RS09325 bp1894019 CTT |
| Insertions | MW_RS09320/MW_RS09325 bp1894017 T |

NRS123.8.30

| **Type** | **Mutation** |
| --- | --- |
| Coding | MW_RS00105 G358S  MW_RS02745 A477P  MW_RS09940 P126L  MW_RS12075 R178H |
| Substitutions | MW_RS09320/MW_RS09325 bp1894019 CTT |
| Insertions | MW_RS09320/MW_RS09325 bp1894017 T |

NRS123.8.31

| **Type** | **Mutation** |
| --- | --- |
| Coding | MW_RS00105 N48K  MW_RS00735 E31*  MW_RS09940 L57F |
| Substitutions | MW_RS09320/MW_RS09325 bp1894019 CTT |
| Insertions | MW_RS09320/MW_RS09325 bp1894017 T |

NRS123.8.32

| **Type** | **Mutation** |
| --- | --- |
| Coding | MW_RS00105 D440Y  MW_RS08160 P41L  MW_RS09935 N291K |
| Substitutions | MW_RS09320/MW_RS09325 bp1894019 CTT |
| Insertions | MW_RS09320/MW_RS09325 bp1894017 T |

NRS123.8.33

| **Type** | **Mutation** |
| --- | --- |
| Coding | MW_RS06920 L41S  MW_RS08130 Y82*  MW_RS09080 M339I  MW_RS09935 S329L |
| Intergenic | MW_RS09685 bp1957557 G |
| Substitutions | MW_RS09320/MW_RS09325 bp1894019 CTT |
| Insertions | MW_RS09320/MW_RS09325 bp1894017 T |

NRS123.8.35

| **Type** | **Mutation** |
| --- | --- |
| Coding | MW_RS00105 G223D  MW_RS02745 T622I  MW_RS02800 I222F  MW_RS08900 S335G  MW_RS09940 G140E |
| Substitutions | MW_RS09320/MW_RS09325 bp1894019 CTT |
| Insertions | MW_RS09320/MW_RS09325 bp1894017 T |

NRS123.8.36

| **Type** | **Mutation** |
| --- | --- |
| Coding | MW_RS00105 L10F MW_RS09940 S7L |
| Substitutions | MW_RS09320/MW_RS09325 bp1894019 CTT |
| Insertions | MW_RS09320/MW_RS09325 bp1894017 T |

NRS123.8.37

| **Type** | **Mutation** |
| --- | --- |
| Coding | MW_RS00105 Q216E  MW_RS02275 K92K  MW_RS02440 A89T  MW_RS09940 R163K |
| Substitutions | MW_RS09320/MW_RS09325 bp1894019 CTT |
| Insertions | MW_RS09320/MW_RS09325 bp1894017 T |

NRS123.8.38

| **Type** | **Mutation** |
| --- | --- |
| Coding | MW_RS00105 A243T  MW_RS02415 Y102D  MW_RS09585 P49S  MW_RS09940 P126S |
| Substitutions | MW_RS09320/MW_RS09325 bp1894019 CTT |
| Insertions | MW_RS09320/MW_RS09325 bp1894017 T |

NRS123.8.40

| **Type** | **Mutation** |
| --- | --- |
| Coding | MW_RS00105 T367N  MW_RS02745 Q468L  MW_RS09940 D134G |
| Substitutions | MW_RS09320/MW_RS09325 bp1894019 CTT |
| Insertions | MW_RS09320/MW_RS09325 bp1894017 T |
| Deletions | MW_RS03060 bp 644615-644618  [MW_RS10245]-[MW_RS10565] bp 2046219-2088820 |
