# Supplementary material for: Effect of genetic background on the evolution of Vancomycin-Intermediate Staphylococcus aureus (VISA)
Source: PeerJ. 2021 Jul 13;9:e11764. doi: 10.7717/peerj.11764 (PMC8284308; doi:10.7717/peerj.11764)
Supplement: Supplemental Information 5 — Deletions greater than 2 bp found in evolved VISA strains in the background NRS70 were catalogued. Mutations were called with breseq. [file peerj-09-11764-s005.docx]

| **Gene** | **Description** | **Size (bp)** |
| --- | --- | --- |
| SA_RS04005 | *degV* | 84 |
| SA_RS14675, SA_RS06275 | Hypothetical protein, *rimP* | 75 |
| SA_RS07900 | *dgkA* | 48 |
| SA_RS10070 - SA_RS10405 | Staphylococcus phage StauST398-4 | 44257 |
| SA_RS00245 | *walK* | 3 |
| SA_RS05215 | *purQ* | 3 |
| SA_RS09410 | Helix turn helix transcription regulator | 281 |
| SA_RS04005 | *degV* | 156 |
| SA_RS05345, SA_RS05350 | Cell wall binding lipoprotein, *pdhA* | 1421 |
