# Supplementary material for: Effect of genetic background on the evolution of Vancomycin-Intermediate Staphylococcus aureus (VISA)
Source: PeerJ. 2021 Jul 13;9:e11764. doi: 10.7717/peerj.11764 (PMC8284308; doi:10.7717/peerj.11764)
Supplement: Supplemental Information 6 — Insertions greater than 2 bp found in evolved VISA strains in the background NRS70 were catalogued. Mutations were called with breseq. [file peerj-09-11764-s006.docx]

| **Gene** | **Description** | **Insertion** |
| --- | --- | --- |
| SA_RS07890 | GTPase era | TTTTCC |
