# Supplementary material for: Effect of genetic background on the evolution of Vancomycin-Intermediate Staphylococcus aureus (VISA)
Source: PeerJ. 2021 Jul 13;9:e11764. doi: 10.7717/peerj.11764 (PMC8284308; doi:10.7717/peerj.11764)
Supplement: Supplemental Information 7 — Deletions greater than 2 bp found in evolved VISA strains in the background NRS123 were catalogued. Mutations were called with breseq. [file peerj-09-11764-s007.docx]

| **Gene** | **Description** | **Size (bp)** |
| --- | --- | --- |
| MW_RS05925 | *stk1* | 45 |
| MW_RS10245 - MW_RS10565 | Staphylococcus phage Pvl108 | 42602 |
| MW_RS03060 | DU443 domain-containing protein | 4 |
| MW_RS14510, MW_RS02905, MW_RS02910, MW_RS02915, MW_RS02920 | Hypothetical protein, *vraA, vraB, vraC,* hypothetical protein | 3842 |
| MW_RS08165, MW_RS08170 | *mtaB*, rRNA small subunit methyltransferase | 2420 |
| Intergenic MW_RS12225 & MW_RS12230 | Intergenic  *hutG, sdpC* | 135 |
| MW_RS03860 | *tagO* | 51 |
