# Supplementary material for: Effect of genetic background on the evolution of Vancomycin-Intermediate Staphylococcus aureus (VISA)
Source: PeerJ. 2021 Jul 13;9:e11764. doi: 10.7717/peerj.11764 (PMC8284308; doi:10.7717/peerj.11764)
Supplement: Supplemental Information 8 — Deletions greater than 2 bp found in evolved VISA strains in the background NRS384 were catalogued. Mutations were called with breseq. [file peerj-09-11764-s008.docx]

| **Gene** | **Description** | **Size (bp)** |
| --- | --- | --- |
| Intergenic NRS_1842 & NRS_1843 | Intergenic  IS1181 transposase, tRNA-leu | 51 |
| NRS_1469 | *srrA* | 5 |
| NRS_1951 - NRS_2005 | Staphylococcus phage 23MRA | 43048 |
| NRS_1650 | *clpX* | 41 |
| NRS_1619 | *relA2* | 30 |
| NRS_2214 | *rpoA* | 15 |
| NRS_2117 | *rpoE* | 126 |
| NRS_2620 | *sasF* | 12 |
