# Supplementary material for: Effect of genetic background on the evolution of Vancomycin-Intermediate Staphylococcus aureus (VISA)
Source: PeerJ. 2021 Jul 13;9:e11764. doi: 10.7717/peerj.11764 (PMC8284308; doi:10.7717/peerj.11764)
Supplement: Supplemental Information 9 — Insertions greater than 2 bp found in evolved VISA strains in the background NRS384 were catalogued. Mutations were called with breseq. [file peerj-09-11764-s009.docx]

| **Gene** | **Description** | **Insertion** |
| --- | --- | --- |
| NRS_261 | *tarL* | CCTGAAGATAAGTACTTA |
| NRS_1202 | *rny* | AAAGAA |
| NRS_1897 | *vraR* | TACGGTTACGCATTTTCA |
| NRS_1939 | DUF1700 domain-containing protein | ACTAAT |
| Intergenic  NRS_2521 & NRS_2522 | Intergenic  *ssaA2_3, mvaA* | TCAG  *Two 1 bp insertions also in this region |
